# Influence of femoral implant design modification on anterior knee pain and patellar crepitus in patients who underwent total knee arthroplasty without patella resurfacing

**DOI:** 10.1186/s12891-020-03391-2

**Published:** 2020-06-09

**Authors:** Yi-Fan Huang, Yu-Hang Gao, Lu Ding, Bo Liu, Jian-Guo Liu, Xin Qi

**Affiliations:** Department of Orthopaedic Surgery, The First Hospital of Jilin University, Changchun, Jilin, 130021 China

**Keywords:** Total knee arthroplasty, Femoral component, Prosthesis design, Anterior knee pain, Patellar crepitus

## Abstract

**Background:**

The incidence of patient dissatisfaction due to anterior knee pain (AKP) and patellar crepitus after total knee arthroplasty (TKA) remains a concern. However, it has been shown that improvements in the femoral component of traditional prostheses could reduce these instances of pain in the case of TKA performed with patellar resurfacing. This study aims to investigate whether TKA without patellar resurfacing can also benefit from the aforementioned femoral component modification in reducing AKP and patellar crepitus post-TKA.

**Methods:**

Sixty-two patients (85 knees) who underwent TKA using the modern prosthesis and 62 age- and sex-matched patients (90 knees) fitted with the traditional prosthesis were enrolled in this study. The occurrence of AKP and patellar crepitus as well as the Knee Society Score (KSS) were consequently recorded, and the data was analyzed in order to determine whether there was a statistically significant difference between the two groups.

**Results:**

The incidence of AKP was significantly lower in the study group compared with the control group at the 3-month and 1-year follow-ups (4.7% vs. 13.3% [*p* = 0.048] and 3.5% vs. 13.3% [*p* = 0.021], respectively). In addition, the incidence of patellar crepitus was also significantly lower in the study group compared with the control group at the 3-month and 1-year follow-ups (15.3% vs. 34.4% [*p* = 0.004] and 10.6% vs. 28.9% [*p* = 0.002], respectively). There was no significant difference in the KSS between the two groups.

**Conclusions:**

These results revealed that TKA without patellar resurfacing will indeed benefit from the modified femoral implant design in reducing AKP and patellar crepitus, a finding that may be beneficial to surgeons who select implants for their patients when patellar resurfacing is not planned or not possible due to other reasons.

## Background

Although good clinical outcomes have been achieved in patients undergoing total knee arthroplasty (TKA), the incidence of patient dissatisfaction due to multiple factors, especially anterior knee pain (AKP) and patellar crepitus after TKA, remains a concern [1–3]. The etiology and pathogenesis of AKP after TKA remain unclear, although several contributing factors have been identified, like patellar instability and maltracking [4, 5]. Patellar crepitus is defined as a grinding sensation in the region of the distal quadriceps tendon over the patella when the knee is brought from flexion to extension, and secondary to fibrosynovial proliferation on the posterior aspect of the distal quadriceps tendon [6]. A previous study reported that up to one-third of patients who underwent TKA experienced mild to moderate AKP at the 1-year follow-up [7]. The incidence of patellar crepitus in posterior stabilized TKA can reach 45%, which was significantly higher than in other types of prosthesis procedures [8]. Currently, new prostheses have incorporated modifications in the femoral component, with the aim of reducing complications, especially AKP and patellar crepitus. The main modifications of the new prostheses incorporate a new design of the femoral component that is more “friendly” to the patella, including a deeper and more extensive femoral trochlear groove and a smoother intercondylar box transition zone [9]. Recent clinical studies have shown that the femoral implant design modification in the ATTUNE Knee System (DePuy, Inc., Warsaw, IN, USA) decreases the incidence of AKP and patellar crepitus in posterior stabilized TKA [10, 11]. Carey et al. even suggested that a significant difference was demonstrated in the 6 month postoperative clinical outcome between the P.F.C. (DePuy, Inc., Warsaw, IN, USA) and ATTUNE Knee Systems in patients who underwent TKA with both prostheses [12]. However, these studies did not enroll patients who underwent TKA without patellar resurfacing. Although many studies have suggested there is no significant difference in functional outcomes between TKA procedures that incorporated patellar resurfacing and that those that did not [13–15], the probability of AKP was higher in TKA procedures performed without patellar resurfacing than in those that were [16]. Whether TKA without patellar resurfacing benefits from this femoral implant design modification with regard to AKP and patellar crepitus remained unclear. The purpose of the present study, therefore, was to compare the incidence of AKP and patellar crepitus between patients who underwent TKA without patellar resurfacing using either the P.F.C. Sigma (DePuy Synthes) or the ATTUNE Knee Systems. Follow up data were taken at the 3 month and 1 year mark, postoperatively.

## Materials and methods

### Study design

A prospective, nested case-control study was performed in the authors’ department, in accordance with the principles of the Helsinki Declaration. The Institutional Review Board (IRB) approved the study (IRB number IRB00008484), and all patients provided informed consent for the treatment and publication of their anonymized data.

### Participants

All patients scheduled for primary TKA for end-stage knee osteoarthritis between August 2016 and July 2017 were enrolled in this study. Individuals with constrained implant(s), revision, infection, postoperative disability caused by other serious diseases, and those lost in follow-up were excluded. Patients using the modern prosthesis system (i.e., ATTUNE) were selected as the study group. In creating the control group, we matched each study patient (ATTUNE group) with a patient using the traditional prosthesis (i.e., P.F.C. Sigma) for age (±3 years) and gender. Both prostheses are fixed bearing posterior stabilized total knee prostheses. Data on demographic characteristics and Knee Society Score (KSS) [17] were collected preoperatively for all participants.

A single senior surgeon performed all TKAs using a medial parapatellar approach. The anterior and posterior cruciate ligaments were removed. After proximal tibial and distal femoral bone cutting, a spacer was used to evaluate the extension gap to obtain a rectangular and equal extension gap. A stepwise release strategy was applied according to the tension of the soft tissue and ligaments. If the imbalance persisted, a subperiosteal peel method was used to further release. Femoral component rotation was determined using the gap balancing method. In 90° flexion, a tension balancer that rotated on the axis of the femoral medullary canal was placed, and a line was drawn on the posterior femur that created a rectangular flexion gap of the same thickness as the extension gap. External femoral osteotomy was performed according to this gap balance line. An extramedullary device was used to determine the rotation of the tibial component. The proximal anatomical marker was the line connecting the medial border of the tibial tuberosity with the center of the posterior cruciate ligament, and the distal marker was the second metatarsal. The patella was reshaped to remove osteophytes. All knees exhibited good patellar tracking. In addition, patellar cartilage defect(s) were graded intraoperatively according to previous literature reports [18]. An identical postoperative rehabilitation program was scheduled for all patients.

Patients were evaluated at 3 months and 1 year after surgery, with radiographs acquired during each evaluation. KSS scores, AKP, and patellar crepitus were recorded by an investigator who was blinded to the prosthesis type. AKP was diagnosed if pain in the front of the knee was reported, and the degree of pain was scored using a visual analog scale (VAS), as follows: 0 or 1 = no pain; 2 or 3 = mild pain; 4–6 = moderate pain; 7 or 8 = severe pain; and 9 or 10 = excruciating pain. A closed-chain weight-bearing exercise was used, and a series of further questions was administered to those who experienced AKP, as follows: During which of the following actions do you feel pain?: at rest; walking on flat ground; ascending and descending stairs; and squatting. The investigator diagnosed patellar crepitus by placing his hand on the patient’s peripatellar region during full extension to flexion.

### Statistical methods

Data from the two groups were analyzed statistically. Data normality was evaluated using the Kolmogorov-Smirnov test. Independent samples *t*-tests were applied to normally distributed values, while data with non-Gaussian distribution were analyzed using the non-parametric Mann-Whitney *U* test. The observed frequencies of categorical variables were assessed using Pearson’s chi-squared test. Descriptive statistics are expressed as mean with standard deviation and frequencies with percentages for categorical variables. All statistical analyses were performed using SPSS version 19 (IBM Corporation, Armonk, NY, USA), and differences with a *p* value < 0.05 were considered to be statistically significant.

## Results

During the recruitment period, 202 consecutive patients were approached. There were 6 with constrained implant for severe varus or valgus deformity, 1 who died of unrelated disease, and 15 who were lost during follow-up that were excluded. Therefore, 180 patients were eligible for enrollment, of whom 62 (85 knees) were selected for the study group (23 were bilateral and 39 were unilateral involvements). From the remaining 118 patients, 62 (90 knees) age-matched and sex-matched patients were selected to afford a 1:1 match as the control group. Of these 62 patients, 28 were bilateral and 34 were unilateral involvements.

Demographic data and patellar cartilage grades are summarized in Table 1. There was no significant difference in age (*p* = 0.516), side (*p* = 0.362), body mass index (*p* = 0.878) or patellar cartilage grade (*p* = 0.670) between the two groups. In addition, no evidence of prosthesis loosening or osteolysis was found on postoperative radiographs.

**Table 1:** Patient demographics and patellar cartilage grade

Measure	Control Group	Study Group	*p* value
Patients	62	62	**–**
Knees	90	85	**–**
Gender			**–**
Male	10	10	
Female	52	52	
Side			0.362^a^
unilateral	34	39	
bilateral	28	23	
Age	64.98 ± 4.79	65.06 ± 4.42	0.516^b^
BMI	26.13 ± 2.78	25.81 ± 2.82	0.878^c^
Follow-up	1.0 ± 0.44 (0.9–1.1)	1.0 ± 0.53 (0.9–1.1)	0.226^c^
Patellar cartilage grade	2.16 ± 0.729	2.26 ± 0.676	0.670^c^

^a^Pearson Chi-squared test

^b^Independent-samples t-test

^c^Non-parametric Mann–Whitney U test

There was no significant differences in clinical and functional scores between the study and control groups before surgery, at 3-months, or at the 1-year follow-up (Table 2).

**Table 2:** Preoperative and Postoperative KS clinical and function scores between the two groups

Measure	Control Group	Study Group	*p* value
Clinical scores			
Preoperatively	56.81 ± 12.58	56.13 ± 13.67	0.286^a^
3-m Postoperatively	86.65 ± 7.43	87.77 ± 7.43	0.855^a^
1-y Postoperatively	90.24 ± 7.68	91.19 ± 6.84	0.380^a^
Function scores			
Preoperatively	34.03 ± 11.83	35.24 ± 12.03	0.883^a^
3-m Postoperatively	80.81 ± 10.64	82.34 ± 11.08	0.501^a^
1-y Postoperatively	87.10 ± 8.02	88.55 ± 8.70	0.825^a^

^a^Non-parametric Mann–Whitney U test

The incidence of AKP was significantly lower in the study group compared with the control group at the 3-month and 1-year follow-ups. Similarly, the incidence of patellar crepitus was significantly lower in the study group compared with that in the control group at the 3-month and 1-year follow-ups (Table 3).

**Table 3:** Incidence of anterior knee pain, and patellar crepitus between the two groups

Measure	Control Group	Study Group	*p* value
Anterior knee pain			
3-m Postoperatively	12 (13.3%)	4 (4.7%)	0.048^a^
1-y Postoperatively	12 (13.3%)	3 (3.5%)	0.021^a^
Patellar creptius			
3-m Postoperatively	31 (34.4%)	13 (15.3%)	0.004^a^
1-y Postoperatively	26 (28.9%)	9 (10.6%)	0.002^a^
During which of the following actions do you feel pain?			
Rest	0	0	
Walking on flat	0	0	
Ascending and descending stairs	8	3	
Squatting	12	2	

^a^ Pearson Chi-squared test

No new-onset AKP occurred at 1 year; however, 1 symptomatic knee that underwent TKA with the ATTUNE Knee System became asymptomatic at this time. In the control group, 12 patients reported AKP when squatting, among which 8 reported AKP during ascending and descending stairs. In the study group, 3 knees exhibited AKP when squatting, of which 2 exhibited AKP when ascending and descending stairs (Table 3). Two knees with PFC TKA experienced moderate AKP, which persisted at 1-year postoperatively, the remainder of the cases experienced mild AKP at this time (Table 4).

**Table 4:** VAS of anterior knee pain between the two groups

Measure	none	mild	moderate	severe	excruciating
Control Group					
3-m Postoperatively	0	10 (11.1%)	2 (2.2%)	0	0
1-y Postoperatively	0	10 (11.1%)	2 (2.2%)	0	0
Study Group					
3-m Postoperatively	0	4 (4.7%)	0	0	0
1-y Postoperatively	0	4 (4.7%)	0	0	0

## Discussion

The question of whether or not to resurface the patella in TKA remains controversial [19]. A cost-effectiveness analysis demonstrated the superiority of resurfacing over retention of the patella [20]. On the other hand, selectively not resurfacing the patella appears to yield similar results compared with routine resurfacing [21]. The main feature of the modern prosthesis is the modification of the femoral component, which includes a deeper and more extensive femoral trochlear groove and a smoother intercondylar box transition zone. The new design aims—at least theoretically—to reduce the incidence of patellar crepitus and AKP. Previous research investigating the advantages of the femoral implant design modification only recruited patients who underwent TKA with patellar resurfacing [10–12]. To our knowledge, the present investigation was the first prospective study to compare the incidence of AKP and patellar crepitus in patients who underwent TKA without patellar resurfacing.

A meta-analysis showed that there was no relationship between cartilage condition and AKP [22]. Because this study did not resurface the patella and Vahur Metsna et al. [18] found that the cartilage damage to the patella may be correlated with postoperative AKP, we also matched patellar cartilage grade between our two groups. The clinical outcomes measured according to KSS at the 1-year follow-up were consistent with the outcomes reported by Ranawat et al. [11]. Carey et al. [12] suggested better outcomes at the 6-month follow-up; however, we did not find differences at the 3-month and 1-year follow-ups, which may have been due the different measured scores in the study by Carey et al. (i.e., Western Ontario and McMaster Universities Osteoarthritis Index, Oxford Knee, and Short-form-12 scores), or simply a temporary advantage existed at this period but disappeared at 1 year. Nevertheless, these results may indicate that this femoral implant design modification had no significant influence on clinical outcomes of TKA with or without patellar resurfacing at the short-term follow-up.

Results of this study indicated that the ATTUNE group had a lower incidence of AKP, similar to the study by Ranawat et al. [11]. However, AKP incidences of 3.5 and 13.5% (ATTUNE and P.F.C. Sigma Knee Systems, respectively) were obviously lower than the incidences of AKP reported by Ranawat et al. (12.5 and 25.8%, respectively). Asian patients tends to exhibit a longer duration of knee osteoarthritis. Because prolonged pain often results in an increase in pain tolerance, Asian patients may not be as sensitive to pain as those in developed countries. In previous research involving Japanese patients who underwent TKA, the incidence of AKP was also low (6.5%) [23]. In addition, the incidence of AKP in the ATTUNE group was only approximately one-quarter of that in P.F.C. Sigma group (3.5% vs 13.5%)—this ratio was lower than in the study by Ranawat et al. (12.5% vs 25.8%) [11]. A previous study noted that severe AKP can lead to patient dissatisfaction following primary TKA, and revision was often required [24]. In the present study, no patient underwent secondary patellar replacement. Furthermore, we recorded the state of movement when pain occurred for the assessment of AKP and found that it mainly occurred when ascending and/or descending stairs or squatting. It was rare for an individual to report pain while at rest or walking on flat ground. A sharp increase in pressure on the patella when ascending or descending stairs or squatting may be the underlying reason for a higher level of AKP. We assessed the degree of the pain using a VAS and found that two knees that underwent TKA using the PFC Sigma Knee Systems experienced moderate pain, which had a certain degree of impact on quality of life. In contrast, no patients using the ATTUNE Knee System experienced pain. The remainder of patients who experienced pain in our study reported it to be mild at the 2-year follow-up and it had little impact on the quality of life. These results may indicate that TKA without patellar resurfacing benefits from this femoral implant design modification with regard to AKP.

In the present study, the incidence of patellar crepitus at 1 year (10.6 and 28.9% in ATTUNE and P.F.C. Sigma groups, respectively) was similar to the incidences reported by Ranawat et al. (17.7 and 30.9%, respectively) [11]. In addition, we found that the patellar crepitus occurred at 3 months, postoperatively. A previous analysis of posterior-stabilized TKAs revealed that the mean time to the diagnosis of patellar crepitus was approximately 10 months [25], which may suggest that patellar crepitus occurred at an earlier stage in TKA without patellar resurfacing. However, it does not affect the quality of life of patients given the level of reported satisfaction.

We acknowledge that the present study had several limitations. First, it was not blinded and randomized, so bias cannot be excluded as a confounding variable. However, we managed to prospectively match demographic data between cases and controls. Second, the sample size was calculated using a power analysis because no published data regarding TKA without patellar resurfacing were available for this study. Third, we did not enroll patients who underwent TKA with patellar resurfacing. Future studies should compare AKP and patellar crepitus and include both patients who undergo TKA with or without patellar resurfacing in the same study. Some have suggested that surgical technique, more size options, and rotation of the femoral component may also lead to these differences. As such, further research investigating these factors should be performed [26].

## Conclusions

Modified femoral design is able to reduce postoperative anterior knee pain and crepitus in patients without patellar resurfacing. The finding of the current study is helpful for surgeons to select implants for their patients when patellar resurfacing is not planned or not possible due to other reasons.

## Data Availability

The datasets used and/or analysed during the current study are available from the corresponding author on reasonable request.

## Abbreviations

TKA: Total Knee Arthroplasty
AKP: Anterior Knee Pain
KSSs: Knee Society Scores
VAS: Visual Analog Pain Scores
